# Development of a Quantitative Digital Urinalysis Tool for Detection of Nitrite, Protein, Creatinine, and pH

**DOI:** 10.3390/bios14020070

**Published:** 2024-01-30

**Authors:** Vince S. Siu, Minhua Lu, Kuan Yu Hsieh, Bo Wen, Italo Buleje, Nigel Hinds, Krishna Patel, Bing Dang, Russell Budd

**Affiliations:** 1 IBM T.J. Watson Research Center, Yorktown Heights, NY 10598, USA; minhua@us.ibm.com (M.L.); kuan.yu@ibm.com (K.Y.H.); bwen@us.ibm.com (B.W.); ibuleje@us.ibm.com (I.B.); nhinds@us.ibm.com (N.H.); krishna_radhi1993@yahoo.com (K.P.); dangbing@us.ibm.com (B.D.); rbudd@us.ibm.com (R.B.); 2 Institute of Biomedical Engineering, College of Electrical and Computer Engineering, National Yang Ming Chiao Tung University, Hsinchu 30010, Taiwan; 3 Department of Electrical and Computer Engineering, College of Electrical and Computer Engineering, National Yang Ming Chiao Tung University, Hsinchu 30010, Taiwan

**Keywords:** digital urinalysis, nitrite, protein, creatinine, pH, colorimeter

## Abstract

This paper presents a cost-effective, quantitative, point-of-care solution for urinalysis screening, specifically targeting nitrite, protein, creatinine, and pH in urine samples. Detecting nitrite is crucial for the early identification of urinary tract infections (UTIs), while regularly measuring urinary protein-to-creatinine (UPC) ratios aids in managing kidney health. To address these needs, we developed a portable, transmission-based colorimeter using readily available components, controllable via a smartphone application through Bluetooth. Multiple colorimetric detection strategies for each analyte were identified and tested for sensitivity, specificity, and stability in a salt buffer, artificial urine, and human urine. The colorimeter successfully detected all analytes within their clinically relevant ranges: nitrite (6.25–200 µM), protein (2–1024 mg/dL), creatinine (2–1024 mg/dL), and pH (5.0–8.0). The introduction of quantitative protein and creatinine detection, and a calculated urinary protein-to-creatinine (UPC) ratio at the point-of-care, represents a significant advancement, allowing patients with proteinuria to monitor their condition without frequent lab visits. Furthermore, the colorimeter provides versatile data storage options, facilitating local storage on mobile devices or in the cloud. The paper further details the setup of the colorimeter’s secure connection to a cloud-based environment, and the visualization of time-series analyte measurements in a web-based dashboard.

## 1. Introduction

The advent of the coronavirus disease 19 (COVID-19) outbreak has made telemedicine widely popular as an effective means for medical assessments. The demand for a quantitative, point-of-care (POC) diagnostic tool capable of monitoring key analytes like protein, creatinine, nitrite, and pH in urine has become increasingly crucial. Novel approaches to urinalysis that enable patients to obtain relevant clinical values such as urinary protein-to-creatinine ratios (UPCs) at home align seamlessly with the paradigm shift toward decentralized healthcare and the use of digital health tools to enhance patient care and management. These quantitative POC tools can hold immense potential for outpatient management scenarios, including recurring urinary tract infections (UTIs) or kidney stones, chronic kidney disease, home-based chemotherapy treatments, and proteinuria in pregnancy [1,2].

**Figure 1:**
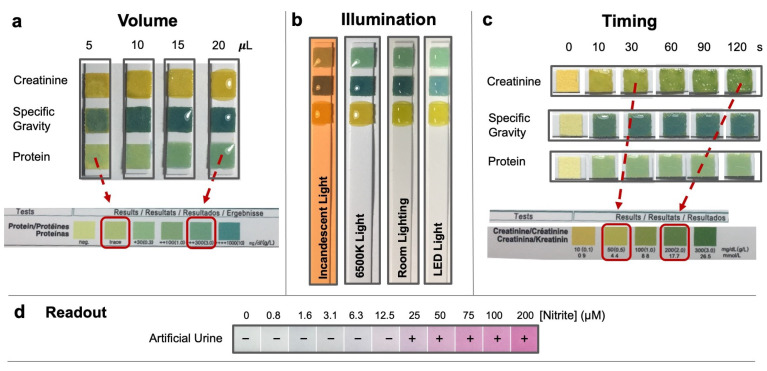
Factors affecting urinary dipstick accuracy. (**a**) Inconsistent liquid volumes on pads can lead to misleading readouts. For instance, a protein dipstick pad dipped in 5 µL versus 20 µL of liquid can display protein concentrations ranging from trace to 300+ mg/dL, as illustrated by the red arrows. (**b**) Variances in illumination can affect color perception. (**c**) Timely readout can impact color interpretation. (**d**) Most urinary dipstick are visually or spectrophotometrically read, providing semi-qualitative data.

Traditional urinalysis relies on subjective dipstick tests. Dipsticks are affordable and disposable, but the readings are subjective and prone to errors, and the accuracy is influenced by several factors [3,4,5,6]. Firstly, proper sample preparation using the dip-and-wipe method is crucial as inadequate or excessive liquid exposure can lead to misleading color changes and inaccurate interpretation (Figure 1a). Secondly, variations in illumination conditions can affect the perceived color gradient of the pads (Figure 1b). Lastly, precise adherence to readout times is necessary for reliable results as the reaction on the dipstick pads continues and evolves over time. Multiple pads on the dipstick have the same readout time, making the simultaneous interpretation of multiple colors challenging, particularly for untrained users (Figure 1c).

The stringent requirements of dipstick urinalysis pose difficulties in achieving accurate in-home testing. Clinical settings use costly reflectance-based analyzers for dipstick reading, providing only semi-quantitative results. For instance, nitrite detection on dipsticks is binary, showing only a positive or negative result for urinary tract infections (UTIs) (Figure 1d) [7]. For protein and creatinine, these analyzers provide readings at fixed levels (e.g., 0, 30, 100, and 200 mg/dL), making it impossible to compute clinical parameters such as the urinary protein-to-creatinine (UPC) ratio. To obtain quantitative readouts, large benchtop chemistry analyzers such as the Roche Cobas^®^ 8000 or the Abbott™ ARCHITECT™ c8000 [8] are used in clinical settings, but these are not suitable for home use.

Efforts to enhance home testing accuracy include initiatives by companies like Healthy.io, Boston, MA, USA [9,10] and Scanwell Health, Los Angeles, CA, USA (now a subsidiary of Becton Dickinson, Franklin Lakes, NJ, USA) [11], use smartphones to capture dipstick images, and interpret colors with built-in algorithms. Other researchers have developed a device that controls for uniform lighting and utilizes smartphone-captured videos to interpret color and results [12,13]. However, these methods still yield semi-quantitative results at best, highlighting the need for a transformative approach in urinalysis to bring quantitative measurements from large clinical chemistry analyzers to a compact form suitable for effective at-home monitoring.

In this paper, we introduce a quantitative, portable, cost-effective, transmission-based colorimeter designed to measure nitrite, protein, creatinine, and pH in urine using four colorimetric assays. A comprehensive performance analysis, presented in Table S1, compares this colorimeter with existing and emerging urinalysis methods. Specifically, our colorimeter outperformed two commercial dipstick analyzers, demonstrating superior nitrite sensitivity, broader protein and creatinine ranges, and more accurate pH readings. The quantitative measurement of protein and creatinine also enables the calculation of the urinary protein-to-creatinine (UPC) ratio, a useful parameter for clinical assessments of chronic kidney diseases and preeclampsia. The colorimeter also offers versatile data storage options, including local storage on mobile phones or in the cloud. The paper outlines a secure setup for connecting the colorimeter to a cloud-based environment and highlights the visualization of time-series analyte measurements through a web-based dashboard. This innovative device represents an initial step towards providing an affordable, portable, and quantitative solution for at-home urinalysis.

## 2. Materials and Methods

### 2.1. Chemicals and Materials

Sulfosalicyclic acid, bovine serum albumin, sodium hydroxide, creatinine hydrochloride, sulfanilamide, N-(1-Naphthyl)ethylene-diamine dihydrochloride (NED), p-arsanilic acid, hydrochloric acid (37%), citric acid, sodium nitrite, methyl red, bromothymol blue, and a nitrite detection kit (Cat. No. 23479) were all purchased from Sigma-Aldrich (St. Louis, MO, USA) and were of analytical grade. 3-hydroxyl-1,2,3,4-tetrahydrobenzo-(h)-quinoline (THBQ) was purchased from Santa Cruz Biotechnology (Dallas, TX, USA). A bicinchoninic acid (BCA) (Cat. No. 23227) and a creatinine assay kit (Cat. No. ab20453) were purchased from ThermoFisher (Waltham, MA, USA) and Abcam (Waltham, MA, USA), respectively. Methanol was purchased from J.T. Baker. Hydrion Buffer Chemvelopes at various pH from 4.0–8.0 were procured from MicroEssential Laboratories (Brooklyn, NY, USA). Artificial urine (Cat. No. 1700-0600) was acquired from Pickering Labs (Mountain View, CA, USA).

### 2.2. Preparation of Reagents for Nitrite Detection

Nitrite detection used the optimized Griess reaction, as described in our previous single-channel colorimeter paper [14]. Briefly, a solution was prepared by adding 0.0325 g of p-arsanilic acid to 24.75 mL of distilled water and then combined with 0.25 mL of hydrochloric acid (37%). This solution was thoroughly mixed, and then 0.0025 g N-(1-Naphthyl)ethylene-diamine dihydrochloride (NED) was added. For the nitrite detection assay, 1 mL of the prepared nitrite detection solution was added to 1 mL of the sample. To assess performance, various concentrations of sodium nitrite were introduced into hydrion salt buffer, artificial urine, and human urine samples.

### 2.3. Preparation of Reagents for Protein Detection

The protein detection reagent solution was prepared by dissolving 1.5 g of sulfosalicylic acid (SSA) in 50 mL of distilled water. After thorough mixing, 0.9 mL of this protein reagent solution was combined with 0.45 mL of the sample. Various concentrations of bovine serum albumin (BSA) were added to the hydrion salt buffer, artificial urine, and human urine samples for testing.

### 2.4. Preparation of Reagents for Creatinine Detection

The creatinine detection reagent solution was prepared by creating a 35 mM picric acid solution and a 0.32 M sodium hydroxide solution. The solutions were mixed in a 1:1 ratio, and 2 mL of the resulting dye mixture was added to 0.2 mL of the sample. Different concentrations of creatinine hydrochloride were introduced into the hydrion salt buffer, artificial urine, and human urine samples for testing.

### 2.5. Preparation of Reagents for pH Detection

The pH detection reagent solution was prepared by combining 0.0012 g of methyl red and 0.016 g of bromothymol blue with 17 mL of ethanol and 23 mL of distilled water. This mixture was diluted 1:10 in distilled water. To measure the pH of the sample, 1 mL of the dye solution was added to 1 mL of the sample.

### 2.6. Colorimeter Measurement

**Figure 2:**
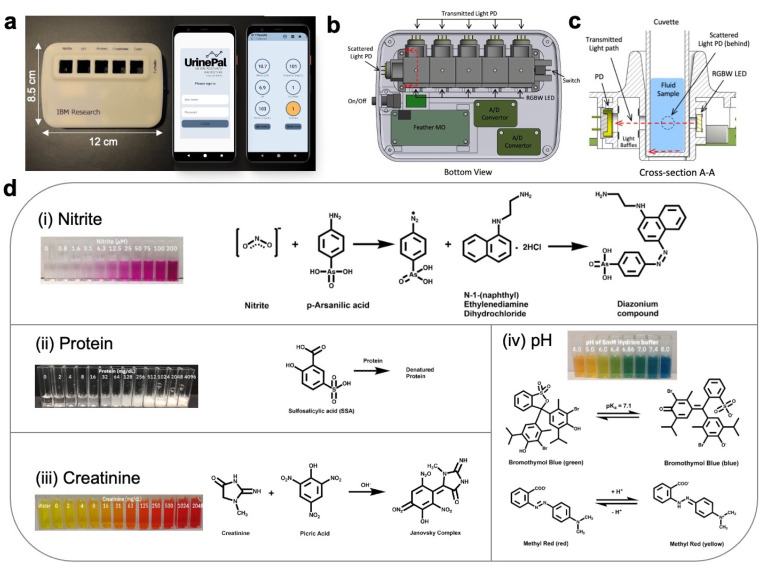
Components and features of the colorimeter. (**a**) Image of the device and its accompanying mobile application. (**b**) Schematic showing top and bottom views and its internal components. The red arrows indicate the cross-section view of the photodetector placement, as denoted by A-A. (**c**) Cross-section view of A-A demonstrating the placement of photodetectors and the paths of transmitted and scattered light. (**d**) Chemical detection reagents for (i) nitrite, (ii) pH, (iii) protein, and (iv) creatinine.

To prepare a sample for measurement, the colorimeter is connected to an Android phone via Bluetooth (Figure 2a). The UrinePal application is then used to perform an initial calibration measurement without sample cuvettes to correct any LED intensity variations. Subsequently, samples with the target analytes are added to the cuvettes with corresponding detection reagents and placed in the appropriate sample slots.

For time-based measurements, the colorimeter measures the sample every 10 s for 10 min. For end-point measurements, 10 readings are taken and averaged at the 10-min mark once the reaction has reached steady-state (Figure S1). The UrinePal application uses the photodetector data, calibration curves, and a color correction algorithm to calculate target analyte concentrations. The results are saved locally in the application and can be transferred to the cloud for further downstream data aggregation, processing, and analysis.

### 2.7. Dipstick Measurement

For comparison, two commercial dipsticks, the Multistix^®^ 10SG (Siemens, Malvern, PA, USA) and Chemstrip^®^ 10+SG (Roche, Indianapolis, IN, USA), were chosen to measure nitrite, protein, and pH. Creatinine was measured with the Multistix^®^ 10SG dipstick. Two reflectance photometers, Clinitek Status+ (Siemens, Malvern, PA, USA) and Urisys 1100 (Roche, Indianapolis, IN, USA), were used to read the dipsticks. The process involved immersing a clean dipstick into a sample, wiping off excess liquid, placing the dipstick on the photometer’s sample tray, and following on-screen instructions. Typically, results were available within 2 min.

## 3. Results and Discussion

### 3.1. Colorimeter Device Design

The multi-channel colorimeter is a handheld device controlled via a mobile phone application over Bluetooth. The device is enclosed in a 115 × 85 × 30 ${\mathrm{mm}}^{3}$ 3D-printed case (Figure 2a) and has five sample slots. The first four slots are for nitrite, pH, protein, and creatinine, while the last one is for color and turbidity assessment. The colorimeter was designed to operate effectively in ambient conditions. The internal components and their layout are visible in the top and bottom views (Figure 2b). Each sample slot has a pair of a multi-wavelength light-emitted diodes (LEDs) and a photodetector on opposing sides for transmission measurements. In the color and turbidity slot, an additional photodetector is set at a 90° angle to capture scattered light (Figure 2c).

The colorimeter uses compact surface-mount red-green-blue-white (RGBW) neopixel LEDs from Adafruit. These LEDs emit light at specific peak wavelengths: red (620–625 nm), green (522–525 nm), blue (465–467 nm), and quasi-white light produced by blending RBG wavelengths [15]. These LEDs are positioned outside the sample holder, with a small aperture for LED exposure. To prevent stray light interference, the photodetector is placed behind a baffle situated 10 mm from the opening. The photodetectors collect transmitted and/or scattered light data, which are then converted into a numerical concentration value using built-in calibration curves for each analyte.

The LEDs and photodetectors are controlled using an Arduino board, like the Adafruit Feather M0 Bluefruit Low Energy board with Bluetooth capabilities for communication with an Android smartphone through a custom-built application developed for data collection, analysis, and storage. The wiring layout of the internal components is illustrated in the electronics diagram (Figure S2).

Liquid samples are measured using 10 mm pathlength polymethyl methacrylate (PMMA) cuvettes containing tailored reagent formulations for specific analyte detection. These cuvettes exhibit an optimum transmission in the visible spectral range from 340 to 800 nm.

### 3.2. Selection of Analyte Detection Reagents

The selection of colorimeter assay reagents for detecting nitrite, protein, creatinine, and pH was based on a comprehensive review of the research literature, patents, urinalysis dipstick, and bench-top clinical chemistry analyzers’ product inserts. The evaluation criteria focused on the ability to detect the target analytes within the clinically relevant range, minimal interference from the sample matrix in both artificial and human urine, and reagent stability for at least 14 days.

Nitrite detection relied on the Griess reaction, consisting of an aniline derivative and a coupling reagent in an acidic solution. The p-arsanilic acid and NED combination proved to be the most sensitive and stable over at least 25 days [14]. A pink azo-dye product resulted from the Griess reaction, leading to maximum absorption in green wavelengths. As such, nitrite concentration was determined using transmitted light intensity from the green LED channel due to its high sensitivity (Figure S3).

For protein detection, albumin, representing 15% of daily urinary protein in healthy individuals, was the target analyte. Various chemical methods, such as tetrabromophenol blue [16,17,18], pyrogallol red [19], benzethonium chloride [20], and sulfosalicylic acid (SSA) [21], were considered. A 3% (*w*/*v*) sulfosalicylic acid solution offered the broadest dynamic sensing range and a simple reagent preparation process. Transmitted and scattered intensities from multiple LED channels exhibited similar sensitivity to protein concentration changes (Figures S4 and S5). The transmitted light intensity from the white LED channel was chosen for calibration and protein concentration determination.

Creatinine detection relied on compounds used in the *Jaffe reaction*, widely adopted as standard reagents in clinical chemistry instruments for blood and urine creatinine measurements [22]. The reaction yields a yellow product at lower creatinine concentrations and an orange-red product at higher concentrations. Both green and blue LED channels showed sensitivity to creatinine concentration changes, with the green channel chosen for calibration and creatinine concentration determination (Figure S6).

For pH measurement, detection reagents optimized specifically for pH values between 5.0 and 8.0 were selected [23,24,25]. The pH solutions displayed varying colors depending on their pH levels. By analyzing transmitted intensities from the red, green, and blue LEDs, the solution’s hue was translated into a corresponding pH value.

### 3.3. Validation of Detection Assay Reagents

**Figure 3:**
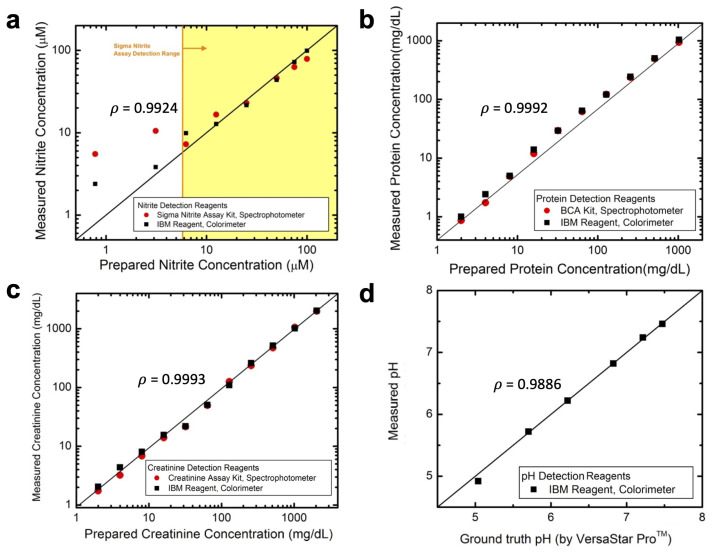
Comparison of colorimeter and gold standard assay measurements for (**a**) nitrite, (**b**) protein, (**c**) creatinine, and (**d**) pH detection in analytes spiked in 5 mM Hydrion Buffer pH 7.0. High Pearson correlation coefficient (ρ) shows a strong positive linear relationship between the gold standard assay and colorimeter measurements. (**a**) is a reprint from reference [14].

In this study, the validation of detection assay reagents for nitrite, protein, creatinine, and pH was conducted to ensure accurate and reliable analyte detection. Nitrite detection reagents were validated against a gold standard kit from Sigma (Cat. No. 23479) using a benchtop spectrophotometer. Correlation analysis demonstrated strong agreement within the gold standard kit’s detection range (6.25–100 µM), with a Pearson correlation coefficient of 0.9924 (Figure 3a). Notably, the colorimeter exhibited higher sensitivity, detecting nitrite as low as 1.6 µM, suggesting enhanced sensitivity compared to the gold standard kit’s Griess reagents.

For protein and creatinine detection, assay reagents were validated against established assays: the bicinchoninic acid (BCA) assay from ThermoFisher (Cat. No. 23227) and the creatinine assay from Abcam (Cat. No. ab20453). Pearson correlation coefficients of 0.9992 and 0.9993 were obtained for protein and creatinine, respectively, indicating strong consistency with gold standard assays (Figure 3b,c).

The pH assay reagents were validated against a calibrated benchtop pH meter (Orion™ VersaStar Pro™ from ThermoFisher Scientific). The colorimeter’s pH measurements demonstrated a strong correlation with the pH meter, confirmed by a Pearson correlation coefficient of 0.9886 (Figure 3d).

### 3.4. Analyte Detection in Artificial Urine

Following assay reagent validation with gold standard assays, the selectivity of the reagents was tested in artificial and human urine samples. Artificial urine samples were prepared by introducing known concentrations of target analytes into a ready-to-use artificial urine solution (Pickering Laboratories, Mountain View, CA, USA) with pH levels varying from 5.0 to 8.0. The composition of artificial urine consists of magnesium, calcium, potassium, ammonium, and iron ions, along with urea, uric acid, lactic acid, and citric acid at typical concentrations found in urine. Successful detection in artificial urine is a first step in demonstrating selectivity of the detection reagents for the target analytes. Calibration curves were generated for nitrite, protein, and creatinine in artificial urine at pH 7.0 (Figure S7). The logistic equations (Equations (1)–(3)) were used to fit the data, establishing relationships between transmitted intensity values, *x*, from the colorimeter and analyte concentrations in the samples.

(1) $$\left[\mathrm{nitrite}\right]=32.59*{\left(\frac{0.99}{\left(x+0.006\right)}-1\right)}^{1/1.68}$$

(2) $$\left[\mathrm{protein}\right]=65.84*{\left(\frac{1.04}{\left(x-0.029\right)}-1\right)}^{1/1.32}$$

(3) $$\left[\mathrm{creatinine}\right]=53.39*{\left(\frac{0.96}{\left(x-0.091\right)}-1\right)}^{1/1.37}$$

For pH determination, the hue value was correlated with known pH values in 10 mM phosphate buffer samples with a pH range of 4.5 to 8.1. For pH values under 5.5, a linear fit (Equation (4)) was used, and for pH values above 5.5, a Boltzmann fit (Equation (5)) was applied.

(4) $$\mathrm{pH}=\frac{\mathrm{Hue}+0.46}{0.21}$$

(5) $$\mathrm{pH}=0.17\times \ln\left(\frac{-2.36}{\mathrm{Hue}-3.16}-1\right)+6.61$$

**Figure 4:**
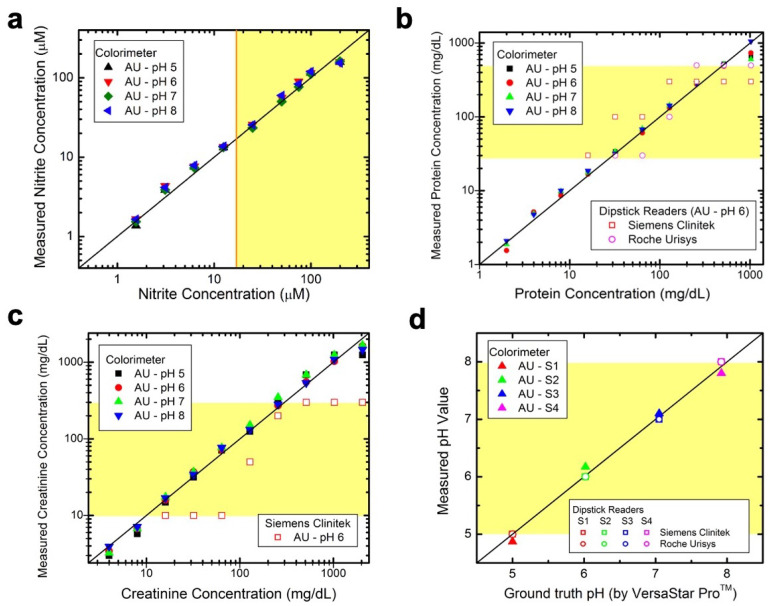
Sensing ranges are depicted for the colorimeter, Siemens Clinitek, and Roche Urisys dipstick analyzers for measuring samples of (**a**) nitrite, (**b**) protein, (**c**) creatinine, and (**d**) pH in artificial urine within the pH range of 5.0–8.0. The yellow boxes in (**a**–**d**) highlight the detection range of the dipstick analyzer(s). (**a**) is a reprint from reference [14].

Samples with known analyte concentrations were prepared in artificial urine and analyzed using these calibration curves. The resulting concentrations showed strong agreement with the known values, indicating accurate detection. The colorimeter effectively measured nitrite (1.6 to 200 µM), protein (2 to 1024 mg/dL), creatinine (2 to 1024 mg/dL), and pH (5.0 to 8.0) in the artificial urine samples (Figure 4).

To assess the sensitivity of the colorimeter, its performance was compared to that of the Siemens and Roche reflectance analyzers. The colorimeter was significantly more sensitive, detecting nitrite concentrations as low as 1.6 µM (∼16× more sensitive) (Figure 4a), compared to the dipstick analyzers 25 µM limit of detection (Figure 1d). For protein and creatinine, the dipstick readers detected protein concentrations in the range of 15 to 500 mg/dL and creatinine concentrations in the range of 10 to 300 mg/dL, as illustrated by the yellow boxes in Figure 4b,c, respectively. In comparison, the colorimeter displayed a broader detection range for both analytes, which could be valuable for monitoring individuals with chronic kidney disease and proteinuria. In pH determination, the colorimeter performed similarly to the dipstick readers, accurately measuring pH values from 5.0 to 8.0 (Figure 4d).

### 3.5. Analyte Detection in Human Urine

**Figure 5:**
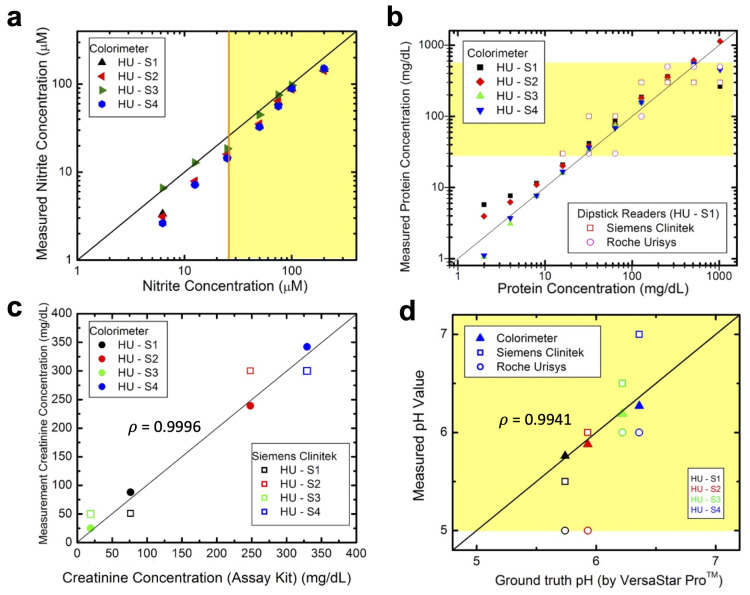
Sensing ranges are shown for the colorimeter, Siemens Clinitek, and Roche Urisys dipstick analyzers for measuring samples of (**a**) nitrite, (**b**) protein, (**c**) creatinine, and (**d**) pH in human urine from pH 5.0–8.0. The yellow boxes in (**a**–**d**) highlight the detection range of the dipstick analyzer(s). (**a**) is a reprint from reference [14].

Similarly, the performance of the colorimeter and the two dipstick analyzers was evaluated in freshly collected human urine. In healthy urine, nitrite and protein should be absent. Nitrite and albumin were spiked into the urine in the ranges of 0–200 µM and 0–1024 mg/dL, respectively, and measured. The colorimeter detected nitrite levels as low as 6.3 µM, approximately four times more sensitive than the dipstick analyzers, which detected nitrite starting at 25 µM. However, slight under-measurement in three of the four human samples suggested possible interference from elements like urobilinogen in the sample matrix (Figure 5a).

Regarding protein detection, the colorimeter measured albumin levels ranging from 4–1024 mg/dL, with minor deviations in the < 10 mg/dL range (Figure 5b), possibly due to trace protein amounts not fully accounted for. For creatinine levels, which vary depending on hydration levels, the colorimeter showed a strong correlation (ρ=0.9996) with a gold standard creatinine assay, enabling precise measurements (Figure 5c). Unlike the semi-qualitative measurements of dipstick analyzers, the colorimeter provided quantitative measurements for protein and creatinine, facilitating the calculation of the urinary protein-to-creatinine (UPC) ratio, which is essential for monitoring patients with chronic kidney health conditions including proteinuria [2].

In pH measurement, the colorimeter exhibited the strongest correlation with a pH meter (ρ=0.9941) compared to the dipstick analyzers (Figure 5d). Bland–Altman plots, which compare the measurement methods, showed the narrowest limits of agreement for the colorimeter, indicating its superior sensitivity and accuracy in measuring pH within the clinically relevant range of pH 5.0–8.0 (Figure S9).

### 3.6. Color and Turbidity Measurements

In addition to measuring nitrite, protein, creatinine, and pH levels in urine, the multiplex colorimeter also assesses the color and turbidity of urine samples. Urine color can vary widely, and it can be influenced by factors such as dehydration, health status, and dietary choices. To address the potential impact of color and turbidity on analyte measurements, we developed correction algorithms detailed in [14]. To demonstrate the color measurement capability, five cuvettes each containing different colored dyes were prepared and inserted into the last channel of the colorimeter. The mobile application displayed the color and turbidity of each solution, represented by a colored circle matching the sample’s hue and a number indicating turbidity, offering valuable insights into the sample’s visual characteristics (Figure S10).

### 3.7. Detection Reagent Stability

Reagent stability is essential for reliable diagnostic assays. The stability of nitrite, protein, creatinine, and pH was tested at room temperature and 4 °C. These reagents were tested with samples containing various target analyte concentrations over 25-days. The results showed that all reagent mixtures remained stable for at least 25 days, regardless of storage temperature (Figure S11). These findings support the practical use of the colorimeter in clinical or at-home settings.

### 3.8. Cloud Connectivity

The colorimeter offers versatile data storage options, both locally on the mobile device and in the cloud. The colorimeter establishes a connection to a cloud-based environment through its companion mobile application. The application’s front-end handles sample measurement. Its back-end consists of the clinical task manager (CTM) that ensures secure data storage in the cloud and expands the application’s capabilities [26], including connecting to physician dashboards for better data visualization, advancing complex data analytics and enabling data sharing with other applications, as well as fostering collaboration, real-time monitoring, and thorough data analysis.

**Figure 6:**
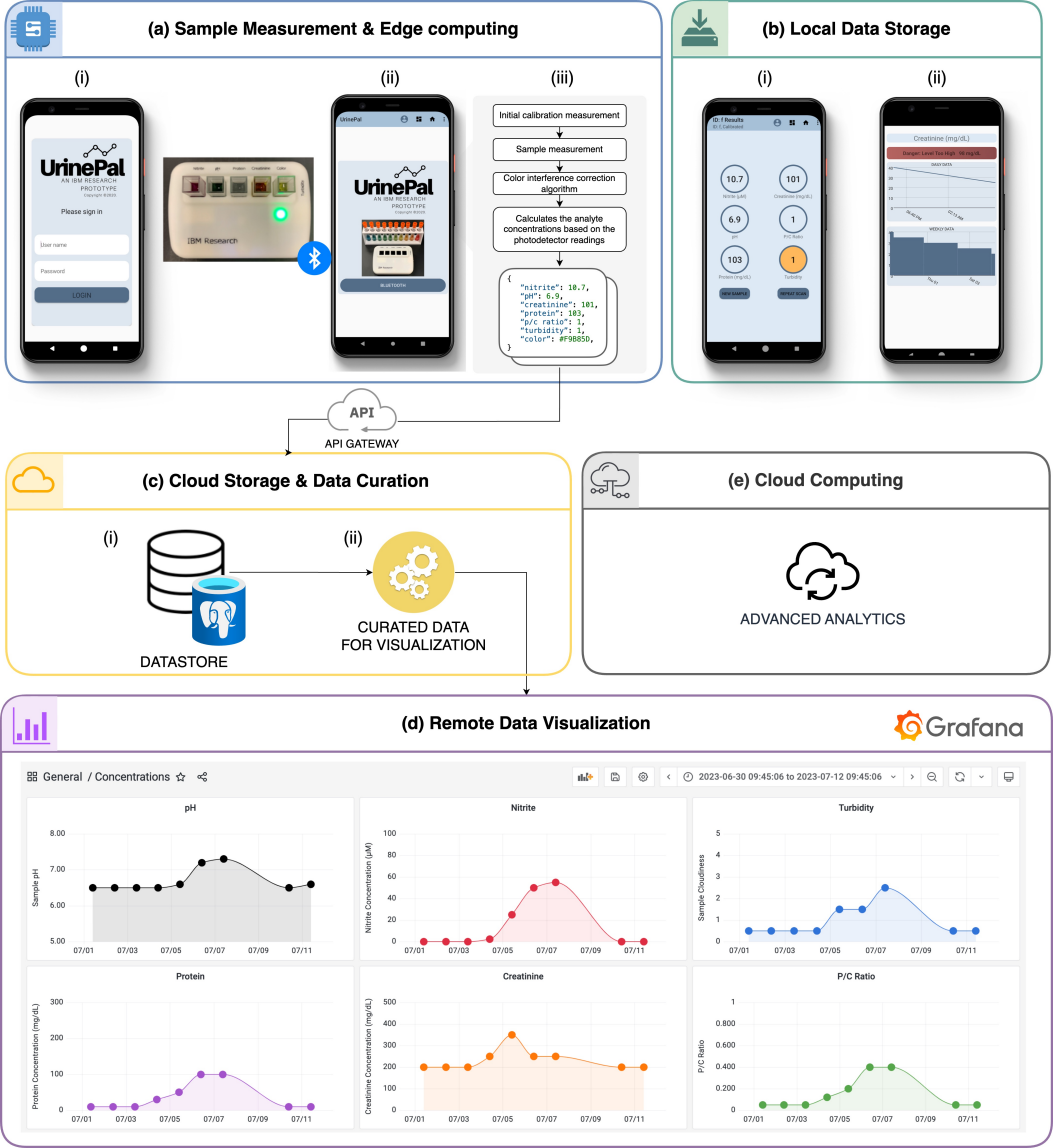
Data architecture. (**a**) Measurement data collected by the colorimeter are transmitted to the mobile application via Bluetooth. Measurement data can be stored either (**b**) locally on the mobile phone or (**c**) passed to a cloud object datastore through an API gateway. (**d**) Access to the cloud-based datastore allows for data visualization and dashboard creation using tools like Grafana, enabling visualization of data trends over time. Each dot in the graphs corresponds to the value of a specific analyte: pH (black), nitrite (red), turbidity (blue), protein (purple), creatinine (orange), and P/C ratio (green), measured on that particular date. (**e**) Data can be combined with other sources to extract meaningful healthcare insights through advanced analytics.

Upon launching the mobile application, users are authenticated by logging in with their credentials (Figure 6a(i)) and assigned a bearer token that is saved for subsequent application programming interface (API) calls. Following the Bluetooth connection to the colorimeter, the device is ready for urine samples measurements (Figure 6a(ii)). After each measurement, the device processes data from the photodetectors and calculates the analyte concentrations. The results are saved on the phone (Figure 6b) as a JSON (Figure 6a(iii)) and sent in an encrypted format via SSL to the back-end through a POST request via an API gateway. The back-end CTM stores the JSON results securely in a PostgreSQL database (Figure 6c(i)).

This architecture facilitates long-term data access and time-series data visualization through seamless integration with a Grafana dashboard linked to the PostgreSQL database. It empowers users to curate data from various timeframes and colorimeter devices (Figure 6c(ii)). With customizable configurations, the dashboard can present data from different analyte measurements within specific timeframes (Figure 6d). This feature offers real-time data access and visualization, making it an invaluable resource for healthcare professionals in long-term patient-monitoring scenarios.

The overall architecture prioritizes user experience while reducing cloud computing costs. The mobile application retains full functionality offline, ensuring uninterrupted usage and batch data upload when the internet connection is re-established. The mobile application also processes data locally, which minimizes delays compared to cloud-dependent analytics prone to data transfer delays [27]. This strategic design reduces network-related issues, increases stability, and optimizes resource usage by moving computational tasks to users’ devices, reducing the need for resource-intensive cloud server processing. This strategic offloading leads to direct cost savings related to server provisioning, data transfer, and cloud computing resource consumption [28].

## 4. Conclusions

In summary, our cost-effective and portable colorimeter offers quantitative measurement of nitrite, protein, creatinine, and pH in urine, enabling at-home urinalysis monitoring. It outperforms dipstick reflectance photometers with a wider sensing range and enhanced sensitivity across all tested analytes. The stability of assay reagents for at least 25 days ensures reliability.

The introduction of quantitative protein and creatinine detection, and a calculated urinary protein-to-creatinine (UPC) ratio at the point-of-care, represents a significant advancement, allowing patients with proteinuria to monitor their condition without frequent lab visits. The colorimeter, coupled with its mobile application featuring cloud connectivity, not only enhances current capabilities but also provides a platform for future clinical expansion.

Future work includes a randomized, controlled clinical trial to compare the performance of the colorimeter outlined in this paper with established clinical instruments such as the dipstck reader, and quantitative large chemistry analyzers, using clinical samples. Another focus is the integration of the colorimeter’s data streaming directly into electronic health records, seamlessly incorporating routine urinalysis into telemedicine practices. Other ongoing efforts involve the expansion of assays to include other relevant biomarkers essential for comprehensive telemedicine assessments. With these advancements, our portable, quantitative colorimeter is poised to transform patient care and monitoring capabilities to be user-friendly and efficient.

## Data Availability

The data presented in this study are available present in this article and its Supplementary Material.

## Supplementary Materials

The following supporting information can be downloaded at: https://www.mdpi.com/article/10.3390/bios14020070/s1, Table S1: Performance attributes of existing and emerging urinalysis methods. Figure S1: Kinetic profiles; Figure S2: Electronic schematic of colorimeter; Figure S3: Transmitted RGBW intensities for nitrite measurements; Figure S4: Transmitted RGBW intensities for protein measurements; Figure S5: Scattered RGBW intensities for nitrite measurements; Figure S6: Transmitted RGBW intensities for creatinine measurements; Figure S7: Calibration curves for nitrite, protein, and creatinine detection; Figure S8: Calibration curves for pH detection; Figure S9: Bland–Altman plots comparing pH measurements; Figure S10: Color measurements on colorimeter; and Figure S11: Reagent stability data.
