# Supplementary material for: Development of a Quantitative Digital Urinalysis Tool for Detection of Nitrite, Protein, Creatinine, and pH
Source: Biosensors (Basel). 2024 Jan 30;14(2):70. doi: 10.3390/bios14020070 (PMC10887154; doi:10.3390/bios14020070)
Supplement: Supplementary file 1 [file biosensors-14-00070-s001.zip › biosensors-2835052-supplementary.pdf]

Supporting Information: Development of a quantitative digital urinalysis tool for detection of nitrite, protein, creatinine, and pH

Vince S. Siu,[*,†,§] Minhua Lu,[†,§] Kuan Yu Hsieh,[†,‡,¶,§] Bo Wen,[†] Italo Buleje,[†] Nigel Hinds,[†] Krishna Patel,[†] Bing Dang,[†] and Russell Budd[†]

†*IBM T.J. Watson Research Center, Yorktown Heights, NY 10598, USA*

‡*Institute of Biomedical Engineering, College of Electrical and Computer Engineering, National Yang Ming Chiao Tung University, Hsinchu 30010, Taiwan*

¶*Department of Electrical and Computer Engineering, College of Electrical and Computer Engineering, National Yang Ming Chiao Tung University, Hsinchu 30010, Taiwan*

§*The authors contributed equally to this work.*

E-mail: vssiu@us.ibm.com

Phone: +1-914-945-2927

Table S1: Performance attributes of existing and emerging urinalysis methods.

	Dipstick	Dipstick and Reflectance Photometer	Dipstick and Smartphone	Colorimeter	Clinical Chemistry Analyzer
Detection Method	Visually by Eye	Reflectance	Reflectance (camera photo)	Transmission	Photometric, Potentiometric, Turbidmetric
Readout	Qualitiative	Semi-quantitative	Semi-quantiative	Quantitative	Quantitative
Device Dimensions (H X W X D)	N/A	6.2” X 6.7” X 10.7”	N/A	3.3” x 4.5” x 1.2”	48” X 79” X 49”
Assays	Nitrite, Protein, Creatinine, pH + others	Nitrite, Protein, Creatinine, pH + others	Nitrite, Albumin, Creatinine	Nitrite, Protein, Creatinine, pH	Nitrite, Protein, Creatinine, pH + others
Nitrite Analytical Sensitivity	Multistix 10SG: 25 μM<br>Chemstrip: 25 μM	Multistix 10SG: 13-21 μM<br>Chemstrip: 10 μM		0.8 μM: 5mM Hydrion salt buffer<br>1.6 μM: Artificial urine	
Nitrite Detection Range	$<$ 25 μM: negative<br>$>$ 25 μM: positive	$<$ 25 μM: negative<br>$>$ 25 μM: positive		0.78 – 200 μM	$<$ 25 μM: negative<br>$>$ 25 μM: positive
Protein Detection Range	30 - 500 mg/dL	30 - 500 mg/dL (quantized)	10 - 150 mg/dL (quantized)	2 – 1024 mg/dL	500 - 18400 mg/dL
Creatinine Detection Range	10 - 300 mg/dL	10 - 300 mg/dL (quantized)	10 - 300 mg/dL (quantized)	2 – 1024 mg/dL	1.35 - 757 mg/dL
Stability	6 months after open bottle	6 months after open bottle		At least 25 days	Varies
Color and Turbidity	No	No	No	Yes	No
Interference Correction	No	No	No	Yes	No
Cost	< $1.00 (dipstick)	$800+ (instrument)<br>< $1.00 (dipstick)	Personal smartphone<br>< $1.00 (dipstick)	< $80 (instrument)	$88,000+ (instrument)<br>$200 to $560 (reagent kit)
Reference	Multistix,[?] Chemstrip[?]	Multistix,[?] Chemstrip[?]	Healthy.io[?] [?]	This work, previous work [?]	Abbott[?] [?] [?]

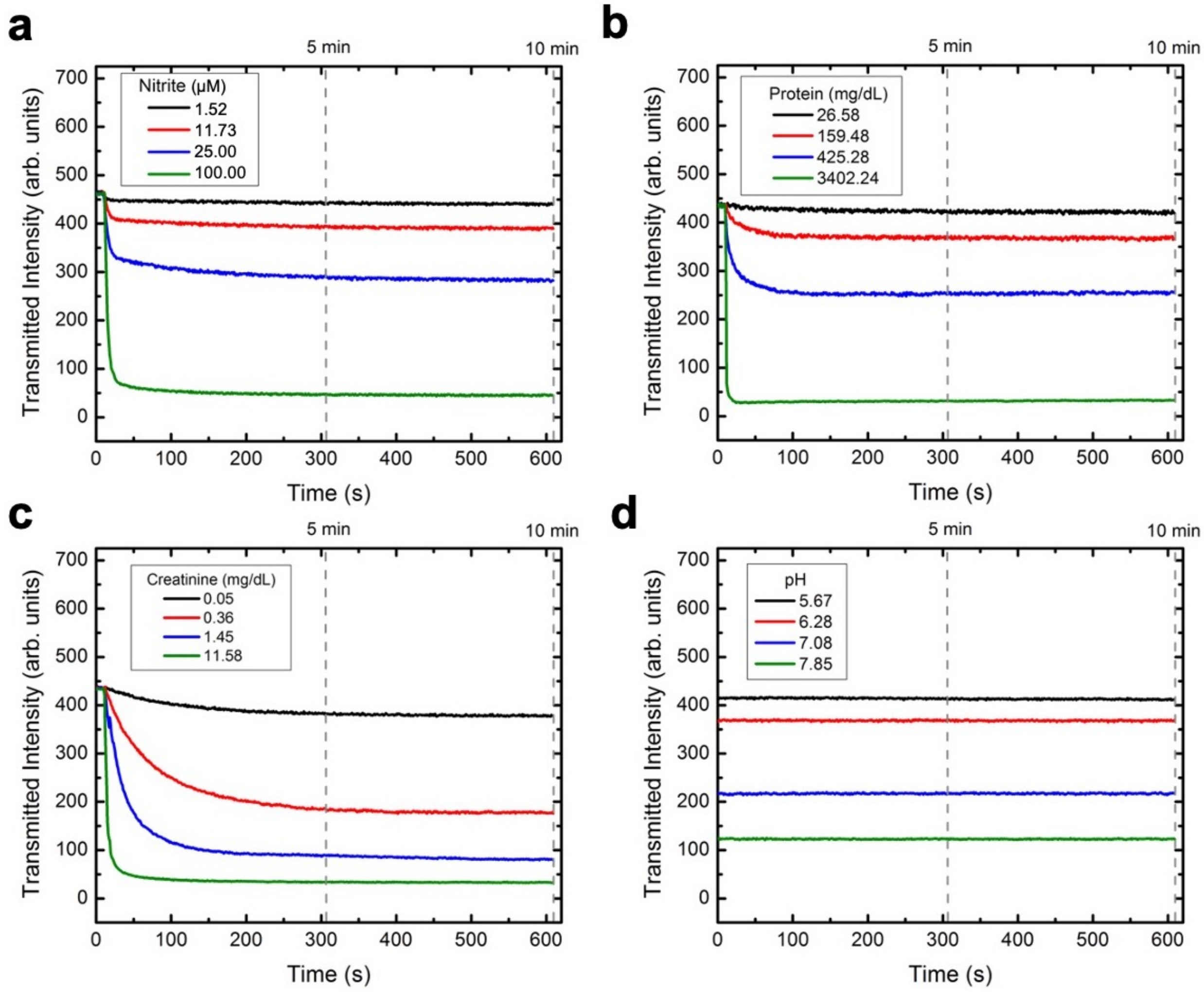

Figure S1: Kinetic profile of samples reacting with a) nitrite, b) pH, c) protein, and d) creatinine reagents at four different concentrations in artificial urine measured over 10 minutes.

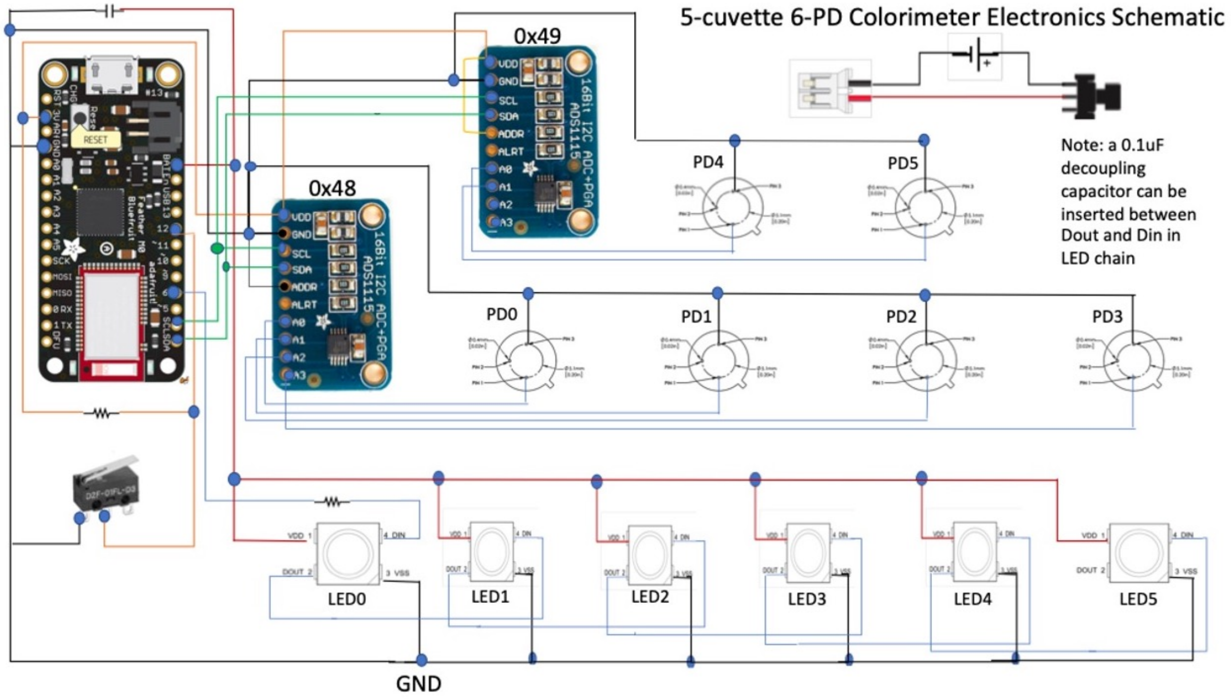

Figure S2: A schematic of a multichannel photometer with 5 sample detection channels. Channels 0 through 3 are transmission only. Channel 4 is equipped with two photodetectors located along the light path and 90 degree from the light path for simultaneous transmission and scattering measurement.

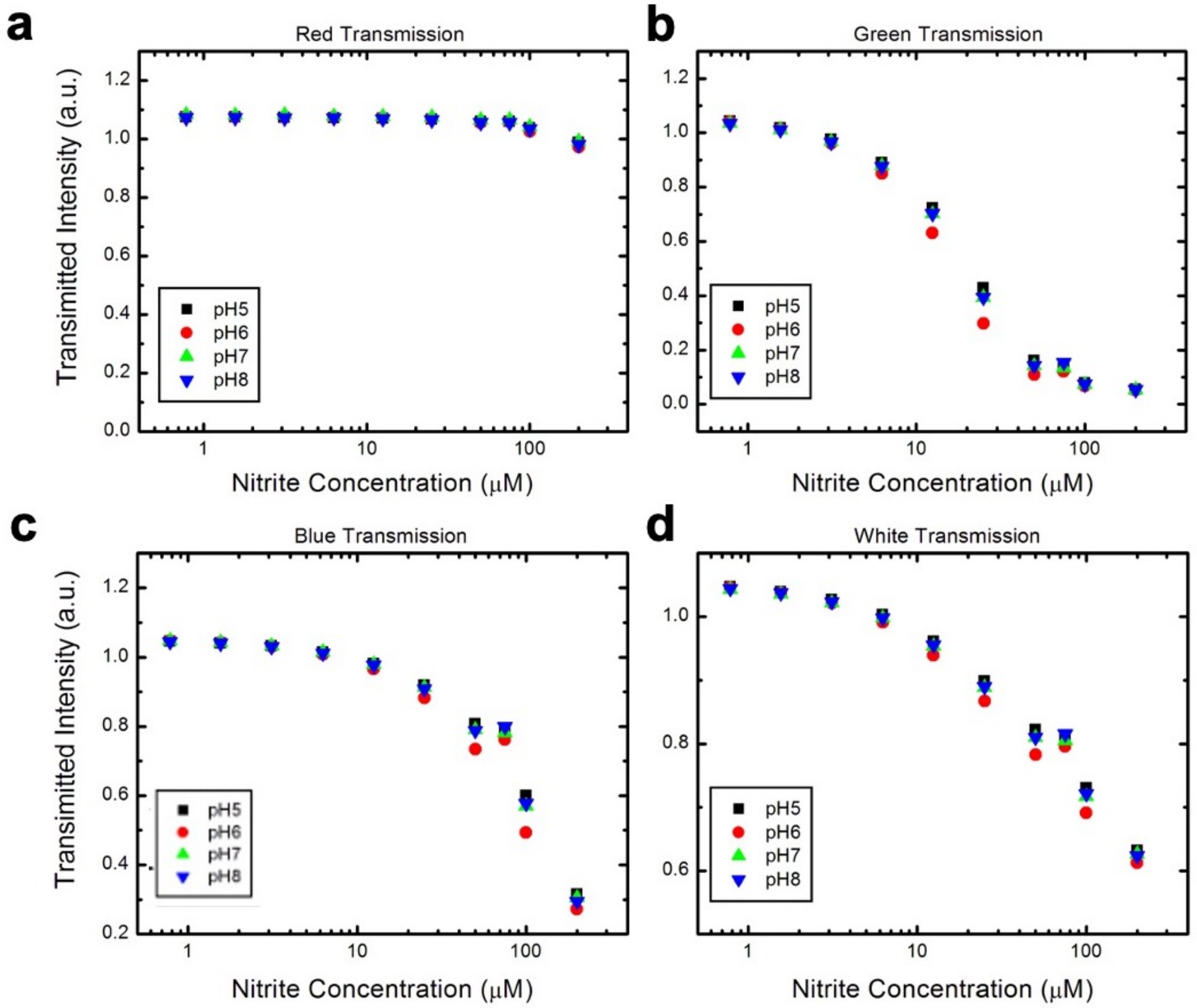

Figure S3: The transmitted intensity of the white, red, green, and blue LEDs measured in samples containing varying concentrations of nitrite and ranging from pH from 5 – 8.

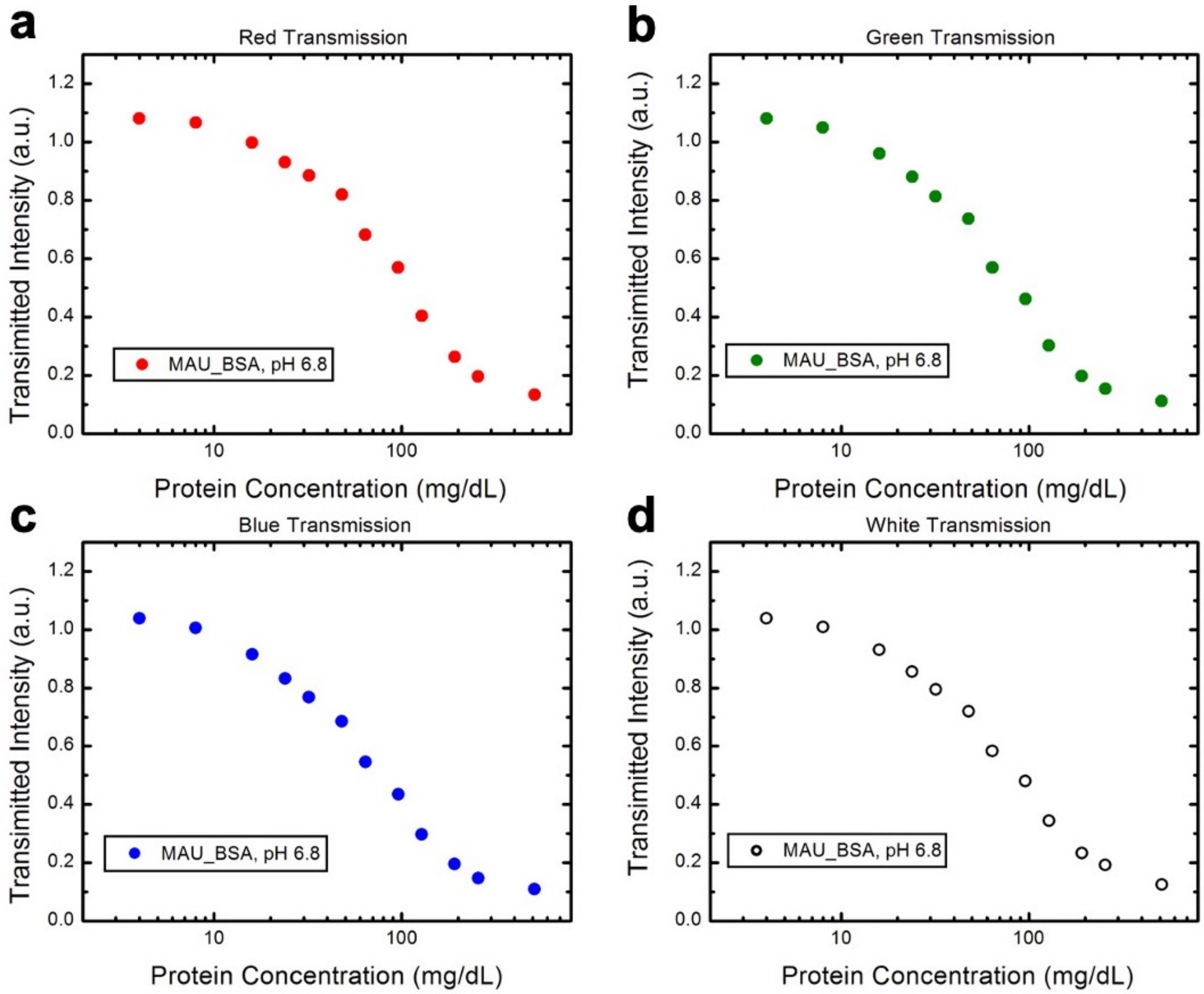

Figure S4: The transmitted intensity of the white, red, green, and blue LEDs measured in samples containing varying concentrations of protein and ranging from pH from 5 – 8.

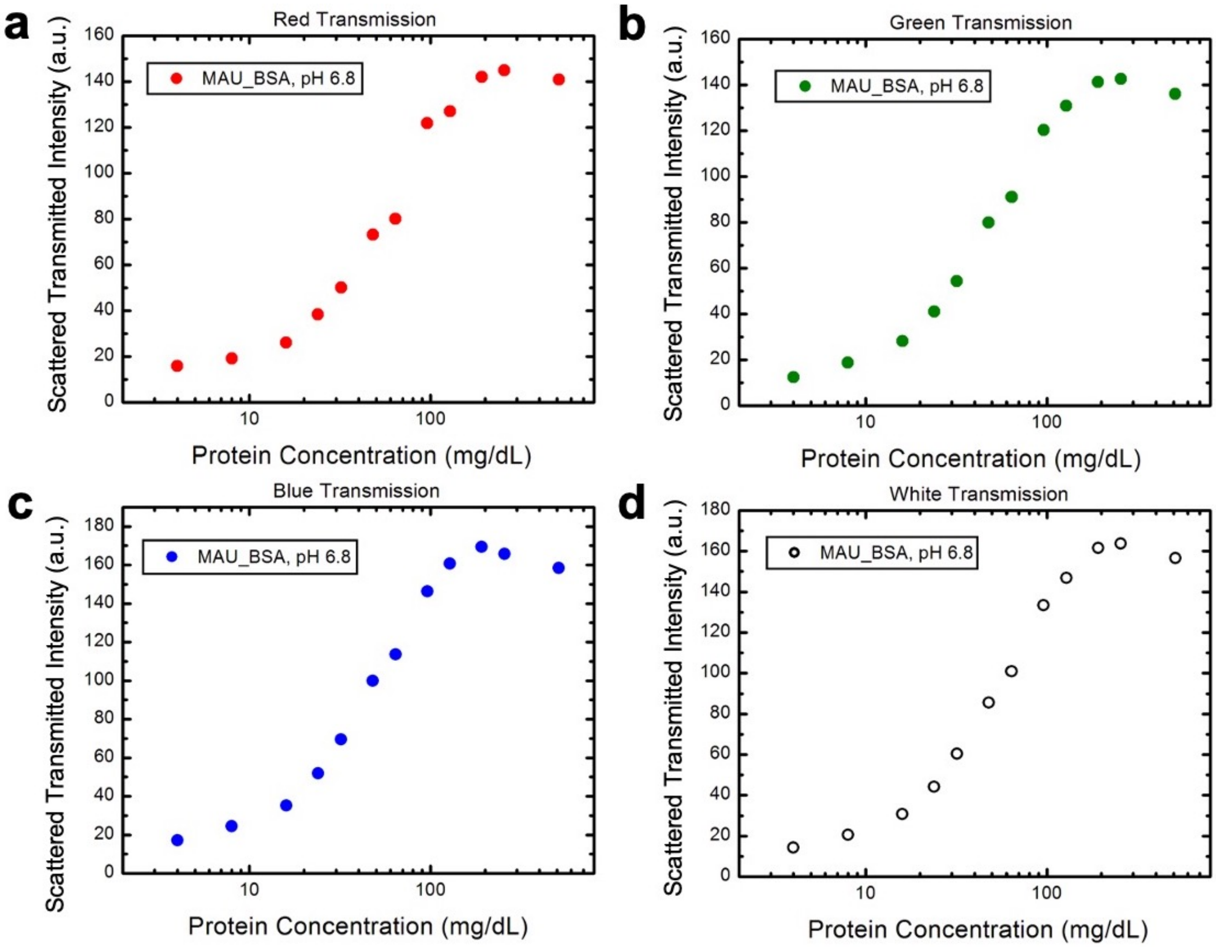

Figure S5: The scattered intensity of the white, red, green, and blue LEDs measured in samples containing varying concentrations of protein and ranging from pH from 5 – 8.

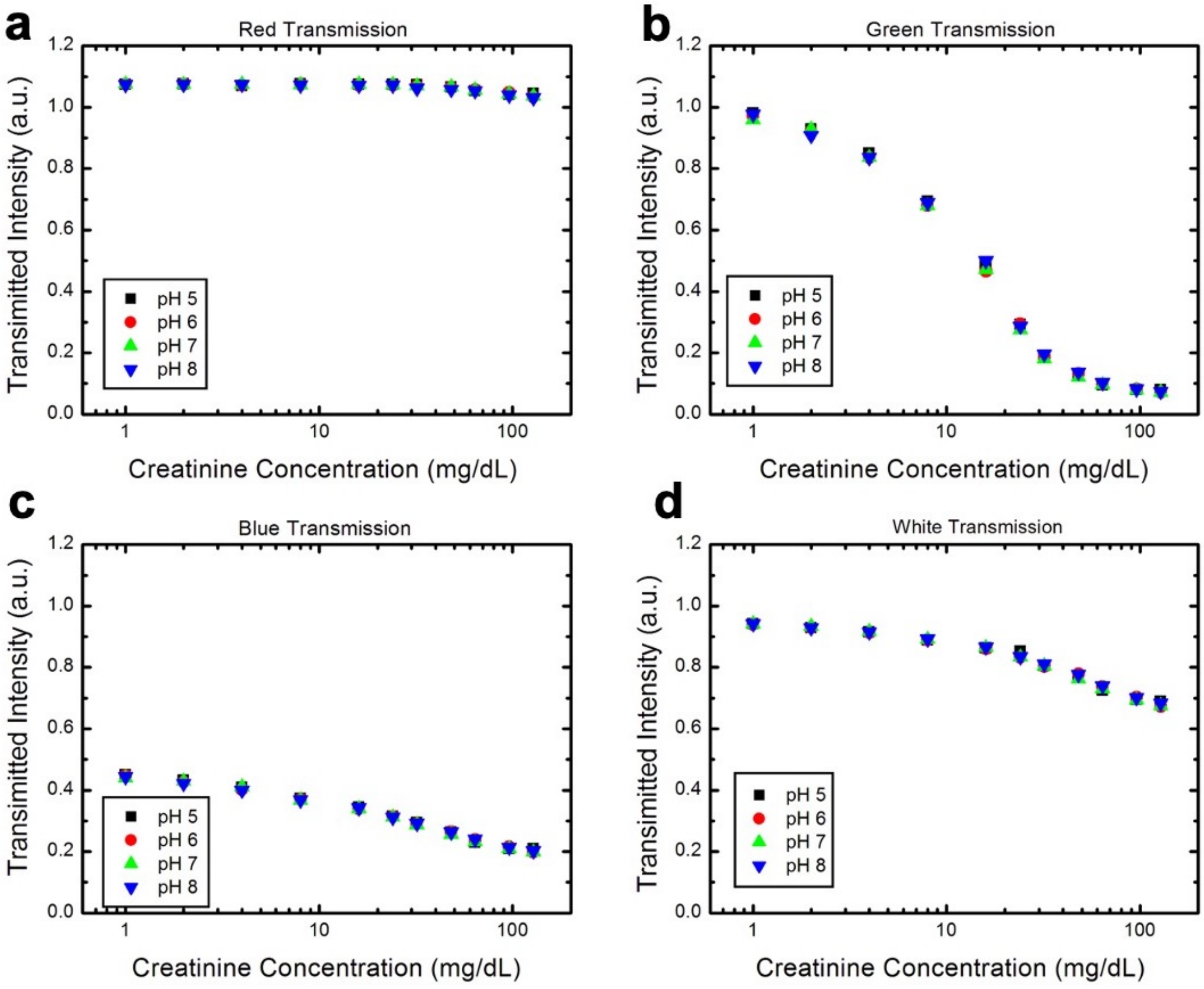

Figure S6: The transmitted intensity of the white, red, green, and blue LEDs measured in samples containing varying concentrations of creatinine and ranging from pH from 5 – 8.

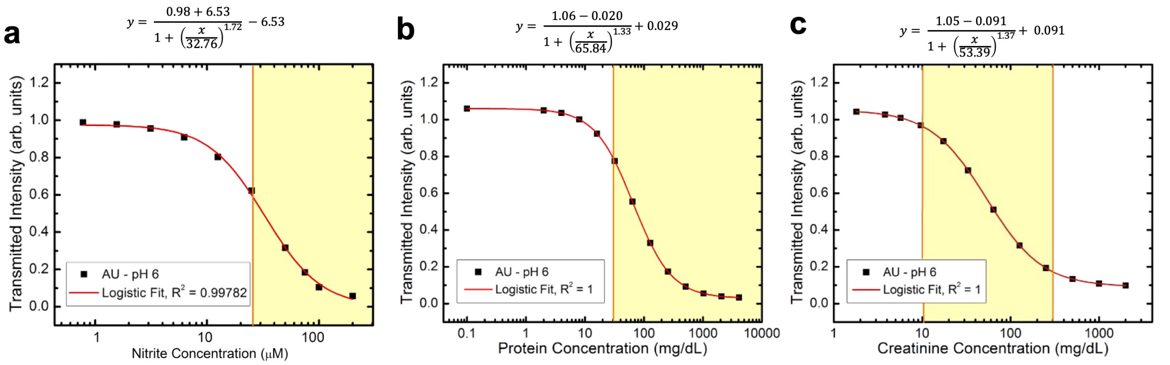

Figure S7: Calibration curve generated for detection of a) nitrite, b) protein and c) creatinine in artificial urine at pH 6.0. The yellow boxes indicate the detection range of the dipstick analyzer.

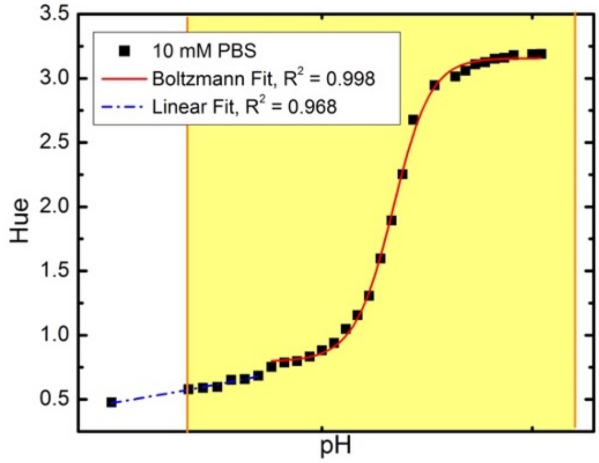

Linear Fit:

$$pH = \frac{(Hue + 0.4618)}{0.2074}$$

Boltzmann Fit:

$$pH = \frac{0.7923 - 3.156}{1 + exp^{(Hue - 6.609)/0.1728}} + 3.156$$

Figure S8: Calibration curve generated for detection of pH in 10 mM phosphate buffer saline (PBS). For pH less than 5.5 (hue values <0.578), a linear fit was used to fit the data, whereas a Boltzmann fit was used to fit the remaining values. The yellow boxes indicate the detection range of the dipstick analyzer.

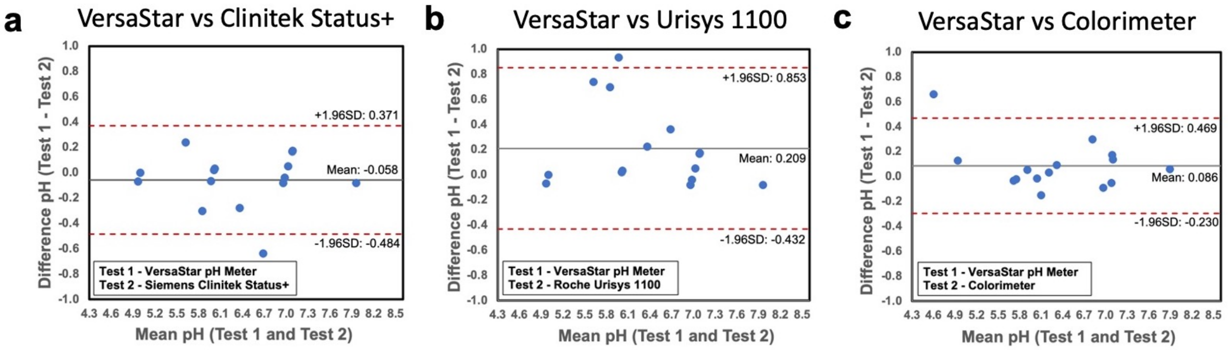

Figure S9: Bland-Altman plots comparing pH measurements in artificial urine samples using a gold standard VersaStar pH meter, comparing with a) Siemens Clinitek Status+, b) Roche Urisys, and c) the colorimeter. Red dotted lines indicate the standard deviation (SD) +/- 0.05%.

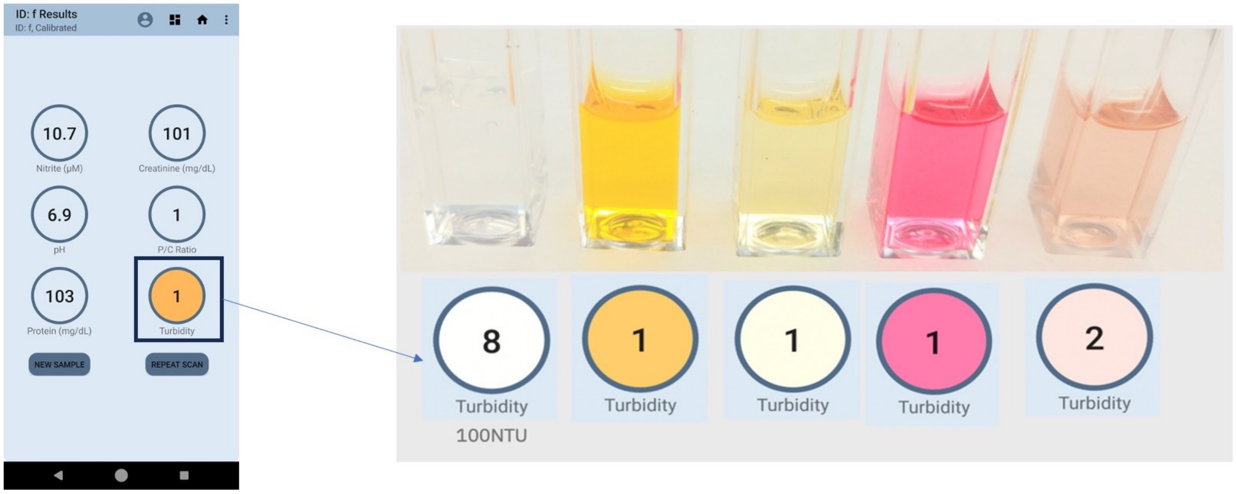

Figure S10: Five cuvettes with different colored dyes were placed in channel 4 of the colorimeter. The color was measured and reported on the app. The color of the circle matches the color of the solution, while the number in the circle is the measurement of turbidity of the sample.

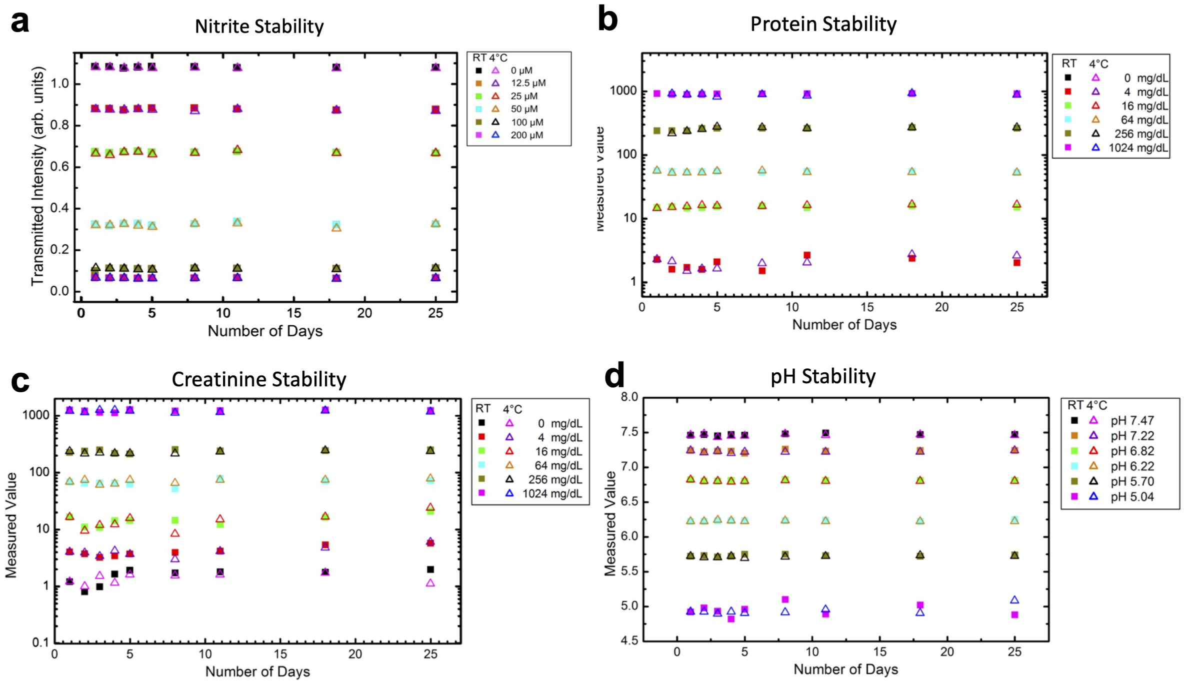

Figure S11: Stability data for a) nitrite, b) protein, c) creatinine, and d) pH detection reagents over a 25 day period.
